# A Novel Efficient FEM Thin Shell Model for Bio-Impedance Analysis

**DOI:** 10.3390/bios10060069

**Published:** 2020-06-17

**Authors:** Jiawei Tang, Mingyang Lu, Yuedong Xie, Wuliang Yin

**Affiliations:** 1 School of Electrical and Electronics Engineering, The University of Manchester, Manchester M13 9PL, UK; jiawei.tang@postgrad.manchester.ac.uk (J.T.); mingyang.lu@manchester.ac.uk (M.L.); 2 School of Instrumentation and Optoelectronic Engineering, Beihang University, Beijing 100036, China; yuedongxie@buaa.edu.cn; 3 Beijing Advanced Innovation Center for Big Data-based Precision Medicine, Beihang University, Beijing 100036, China

**Keywords:** finite element method, thin shell model, β dispersion, Maxwell–Wagner effect, bio-impedance spectroscopy

## Abstract

In this paper, a novel method for accelerating eddy currents calculation on a cell model using the finite element method (FEM) is presented. Due to the tiny thickness of cell membrane, a full-mesh cell model requires a large number of mesh elements and hence intensive computation resources and long time. In this paper, an acceleration method is proposed to reduce the number of mesh elements and therefore reduce the computing time. It is based on the principle of replacing the thin cell membrane with an equivalent thicker structure. The method can reduce the number of mesh elements to 23% and the computational time to 17%, with an error of less than 1%. The method was verified using 2D and 3D finite element methods and can potentially be extended to other thin shell structures. The simulation results were validated by measurement and analytical results.

## 1. Introduction

β-dispersion analysis has been widely investigated for medical [1,2] and industrial [3,4] applications. For example, it has been proposed for the detection of tumours [5,6] and cerebral stroke [7], and it has been applied to the quality inspection of food such as meat, fruit and vegetables [3]. The mechanism of β-dispersion was first described by Schwan [8,9]. β-dispersion takes place at radio frequency, mainly from hundreds of hertz to megahertz. This dispersion is caused by Maxwell–Wagner effect, an interfacial polarisation of cell membrane blocking the ion-flow of intra and extracellular dielectrics.

Analytical solutions are well developed to calculate the β dispersion [10, 11,12]. However, the analytical solution is only designed to analyze the models with a regular shape (i.e., spherical cell model) [13,14]. In reality, most cell shapes are anomalous. The finite element method is a methodology that discretises an entire continuous domain into discrete domains. An irregular cell shape can be simulated in a high accuracy using the finite element method. Therefore, FEM is a feasible and effective way to simulate the dielectric dispersions for models with irregular shapes [15,16].

The difficulty of FEM is that the number of meshing elements is significantly large due to the tiny thickness of the cell membrane; it takes a long time to compute with numerous meshing elements. In this paper, a novel FEM acceleration method was proposed to simulate β dispersion. The acceleration method is to reduce the number of meshing elements and computing time by replacing the full-mesh cell membrane model with an equivalent reduced-mesh model. In this paper, the FEM acceleration method was used to simulate the spherical and oval cell model in two-dimensional and three-dimensional versions.

## 2. Materials and Methods

### 2.1. Single Spherical Cell Model

**Figure 1:**
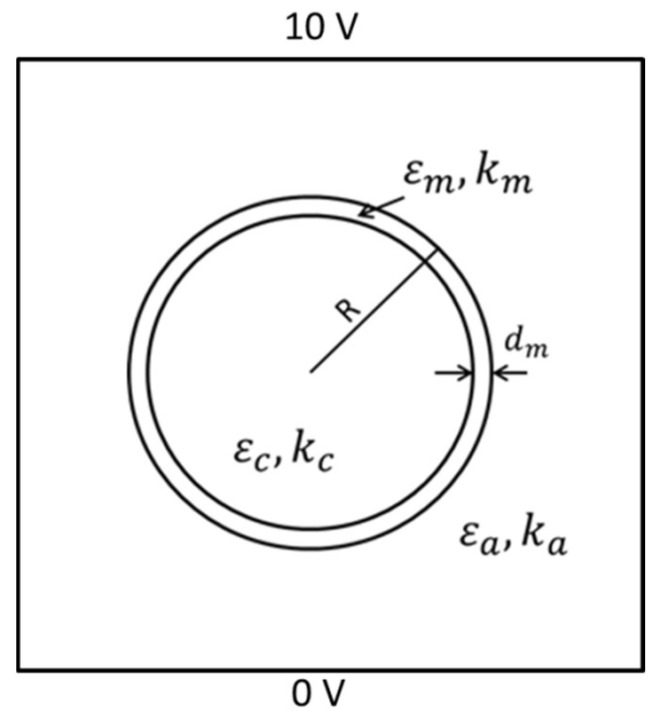
Spherical cell model.

The full-mesh cell model is the single shell spherical model introduced by Asami [15]. The spherical cell model is shown in Figure 1. The parameters of the cell model are $k_{m}=10^{-7}$ S/m, $k_{a}=k_{c}=1$ S/m. $\varepsilon_{c}=\varepsilon_{a}=80$, $\varepsilon_{m}=5$, R = 5000 nm, $d_{m}=5\mathrm{nm}.$ $k_{m}$ is the conductivity of the cell membrane. $k_{a}$ and $k_{c}$ are the conductivity of the extracellular and intracellular fluid, respectively. $\varepsilon_{c}$ and $\varepsilon_{a}$ are the relative permittivity of the intracellular and extracellular fluid, respectively. $\varepsilon_{m}$ is the relative permittivity of the cell membrane. R is the radius of the cell. $d_{m}$ is the cell membrane thickness [10,17,18].

The two-dimensional simulation model is a single cell model, as shown in Figure 1. The spherical cell is put in a suspension with an applied alternating electric field. The voltage of the upper electrode was set to be 10 V, and the voltage of the lower electrode was set to be 0 V [14]. The intracellular and extracellular fluid were conductive while the cell membrane blocked current flow between intracellular and extracellular fluid at low frequency. Dielectric relaxation describes a phenomenon that the permittivity decreases when the frequency of applied electric field increases at a certain range. The relaxation occurs when the electric dipoles are not able to reverse at the same rate with the frequency of the applied field. β-dispersion was observed from 10 kHz to 1 MHz. The dispersion was described by Schwan and could be explained by Maxwell–Wagner effect [8]. The effect occurs at the boundary of layered or inhomogeneous dielectrics. Applying an electric field would build up charges at the boundaries created by different dielectrics. The double-layer dielectrics system can be represented by one capacitance in parallel with one another. Applying a static electric field would charge both capacitances and creates charges at the boundary of the dielectrics. This phenomenon is also called interfacial polarisation.

The equivalent complex relative permittivity of the cell model in Figure 1 can be calculated according to Asami [12]:

(1) $$\varepsilon_{p}^{*}=\varepsilon_{m}^{*}\frac{2\left(1-v\right)\varepsilon_{m}^{*}+\left(1+2v\right)\varepsilon_{c}^{*}}{\left(2+v\right)\varepsilon_{m}^{*}+\left(1-v\right)\varepsilon_{c}^{*}}$$

where v $={\left(1-\frac{d_{m}}{R}\right)}^{n}$, $d_{m}$ is the cell membrane thickness, R is the radius of the cell, and n stands for the factor of dimension. When n = 2, the equation is applied to the two-dimension model. When n = 3, the equation is applied to three-dimension model. $\varepsilon_{p}^{*}$ is the complex relative permittivity of the cell model, $\varepsilon_{m}^{*}$ is the complex relative permittivity of the cell membrane and $\varepsilon_{c}^{*}$ is the complex relative permittivity of cytoplasm; then the complex relative permittivity of the suspension can be calculated by Wagner’s equation:

(2) $$\varepsilon^{*}=\varepsilon_{a}^{*}\frac{2\left(1-P\right)\varepsilon_{a}^{*}+\left(1+2P\right)\varepsilon_{p}^{*}}{\left(2+P\right)\varepsilon_{a}^{*}+\left(1-P\right)\varepsilon_{p}^{*}}$$

where P is the volume fraction, $\varepsilon^{*}$ is the complex relative permittivity of cell suspension, $\varepsilon_{a}^{*}$ is the complex relative permittivity of the external medium.

The conductivity of the suspension can be derived:

(3) $$\varepsilon^{*}=\frac{\sigma^{*}}{{j\omega \varepsilon }_{0}}=\frac{\sigma_{r}+{j\omega \varepsilon }_{r}\varepsilon_{0}}{{j\omega \varepsilon }_{0}}$$

where $\sigma_{r}$ and $\varepsilon_{r}$ are the conductivity and permittivity of the suspension, respectively. $\sigma^{*}$ is the complex conductivity of the suspension, $\varepsilon_{0}$ is the permittivity of vacuum, ω=2πf where f is the frequency of the applied field and j is the imaginary unit.

According to Schwan [9], when $\frac{d_{m}}{R}\ll 1,{\;k}_{m}\ll k_{a}=k_{c},$ the dielectric relaxation of the cell suspension can be represented by the Debye relaxation model:

(4) $$\varepsilon^{*}=\varepsilon_{h}+\frac{\varepsilon_{l}-\varepsilon_{h}}{1+j\omega \tau }+\frac{k_{l}}{{j\omega \varepsilon }_{0}}$$

where $\varepsilon_{h}$ and $\varepsilon_{l}$ are the high-frequency limit and low-frequency limit of the relative permittivity, τ is the relaxation time and $k_{l}$ is the low-frequency limit of the conductivity.

### 2.2. D FEM Simulation

Normally, the finite element problems could be solved by two kinds of methods which are Galerkin’s method and Ritz’s method [19]. In this paper, Galerkin’s method is used to build up simulations.

According to the Gaussian equation,

(5) ∇·D=ρ

where $\vec{D}=\varepsilon \vec{E}$, $\vec{D}$ is the electric flux density, $\vec{E}$ is the electrical field intensity, $\varepsilon =\varepsilon_{r}\varepsilon_{0},$ $\varepsilon_{r}$ is the relative permittivity of the material, ρ is the quantity of electric charge, which is zero in the cell model. So the equation could be rewritten as:

(6) $$\nabla \cdot \left(\sigma +j\omega \varepsilon \right)\vec{E}=\nabla \cdot \left(\sigma +j\omega \varepsilon \right)\nabla U=0$$

where σ is the conductivity of the material and $\varepsilon =\varepsilon_{r}\varepsilon_{0}$. $\varepsilon_{r}$ is the relative permittivity of the material. $\varepsilon_{0}$ is the permittivity of vacuum. U is the electric potential which is the objective value to be solved [20]. To obtain the equivalent conductivity of the total cell suspension, we calculate the total current flowing through the cell suspension from the top electrode to the bottom electrode [15]. According to Kirchhoff’s current law, the current flow through any plane, which is in parallel with the electrodes is the same with the total current flow through the cell suspension. So the current I flow through a chosen plane in parallel with electrodes can be calculated as:

(7) $$I=\sum j^{\left(i\right)}=\sum \sigma^{\left(i\right)}{\vec{E}}^{\left(i\right)}$$

where $j^{\left(i\right)}$ is the current density of the element on the chosen plane, $\sigma^{\left(i\right)}$ is the conductivity of the element on the chosen plane, ${\vec{E}}^{\left(i\right)}$ is the background electrical field on the chosen plane. Then the complex conductivity of the cell suspension can be calculated as:

(8) $$\sigma^{*}=\frac{1}{\rho^{*}}=\frac{L}{S}*\frac{I}{U}$$

where $\sigma^{*}$ and $\rho^{*}$ are the complex conductivity and resistivity, respectively. L is the distance between the top electrode and the bottom electrode. S is the area of the electrodes.

### 2.3. D FEM Simulation

**Figure 2:**
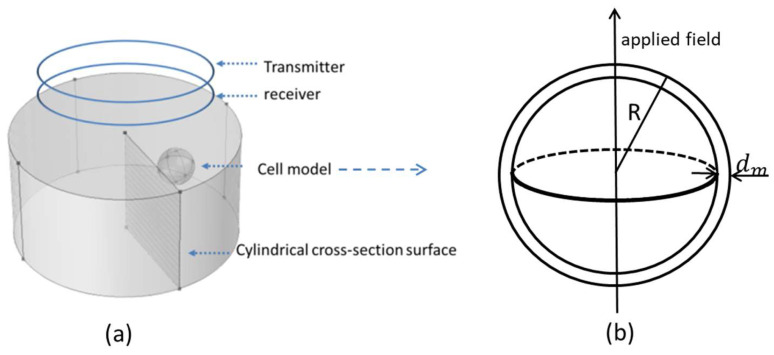
(**a**) 3D suspension model, (**b**) 3D single cell model.

An induction model was built for the three-dimension FEM simulation. As shown in Figure 2, the cylinder stands for the cell suspension, and the spherical model stands for the cell. Imaginary transmitter and receiver coils are put on the top of the suspension to provide an alternating magnetic field as an exciting signal and detect induced secondary magnetic field. The single-cell model in a three-dimension situation is shown in Figure 2b. The applied field is a magnetic field excited by the transmitter in Figure 2a. The radius of the sphere cell model is R and the cell membrane thickness of the sphere cell model is $d_{m}$. All the electric parameters of the sphere cell model are set to be the same with the parameters in Figure 1. The Mxwell–Wagner effect can be calculated by calculating the eddy current distribution induced by the applied magnetic field.

According to Oszkar Biro [21], the basic formulas for the three-dimension edge elements simulation of eddy current problems, Galerkin’s equation is shown as following:

(9) $$\int_{\Omega_{c}}\nabla \times N_{i}\cdot v\nabla \times A^{\left(n\right)}d\Omega +\int_{\Omega_{c}}j\omega \sigma N_{i}\cdot A^{\left(n\right)}d\Omega +\int_{\Omega_{c}}{\sigma N}_{i}\cdot \nabla V^{\left(n\right)}d\Omega =\int_{\Omega_{c}}\nabla \times N_{i}\cdot v_{0}\nabla \times A_{s}d\Omega \;i=1,2,\ldots ,6$$

(10) $$\overset{}{\underset{\Omega_{c}}{\int }}j\omega \sigma \nabla L_{i}\cdot A^{\left(n\right)}d\Omega +\overset{}{\underset{\Omega_{c}}{\int }}\sigma \nabla L_{i}\cdot \nabla V^{\left(n\right)}d\Omega =0\;i=1,2,\ldots ,4$$

where $L_{i}$ is the elemental interpolation of $i^{\mathrm{th}}$ node corresponding to the nth element of it; $N_{i}$ stands for the vector interpolation of $i^{\mathrm{th}}$ edge relating to the nth edge element of it; $A^{\left(n\right)}$ stands for the excited edge vector potential of the nth element; $A_{s}$ represents the original edge vector potential of the nth element; ${\;V}^{\left(n\right)}$ represents the electrical potential excited by the nth element on the receiving coil; v stands for the reluctivity of the model; $v_{0}$ is the reluctivity of the air; σ is the conductivity of the model [22,23,24].

The eddy current density is:

(11) $$J^{\left(i\right)}=\sigma^{\left(i\right)}E^{\left(i\right)}$$

where $E^{\left(i\right)}$ denotes the vector sum of the electrical field on all the edges of each tetrahedral element, $\sigma^{\left(i\right)}$ is the complex conductivity parameter of the of each tetrahedral element.

The model is shown in Figure 2. Assuming there is an equivalent model with uniform dielectric and the background suspension has exactly the same shape with the simulation model in Figure 2. Since the normal component of the electrical field relative to each cross-section surface of suspension (shown in Figure 2) is identical, then

(12) $$\sum \vec{n}\cdot E^{\left(i\right)}=\sum \vec{n}\cdot E_{s}^{\left(i\right)}$$

where, $E_{s}^{\left(i\right)}$ denotes the background electrical field of the of each tetrahedral element; $\vec{n}$ is the normal unit vector relative to the surface of target; 

Since the equivalent model is uniform, the electrical background field $E_{s}^{\left(i\right)}$ is always vertical to every cylindrical cross-section surface of the suspension model shown in Figure 2. Then the equivalent complex conductivity of the original suspension (as shown in Figure 2) can be derived from (8) and (9) that

(13) $$\sigma_{s}=\frac{\sum J_{s}^{\left(i\right)}}{\sum \vec{n}\cdot \frac{J^{\left(i\right)}}{\sigma^{\left(i\right)}}}$$

$\sigma^{\left(i\right)}$ is the complex conductivity of each tetrahedral element, $J_{s}^{\left(i\right)}$ is the eddy current density through each cylindrical cross-section of equivalent suspension model, $J^{\left(i\right)}$ is the eddy current density through each cylindrical cross-section of the original suspension model with arranged cells and $\vec{n}$ is the current flow direction.

### 2.4. Acceleration Method

**Figure 3:**
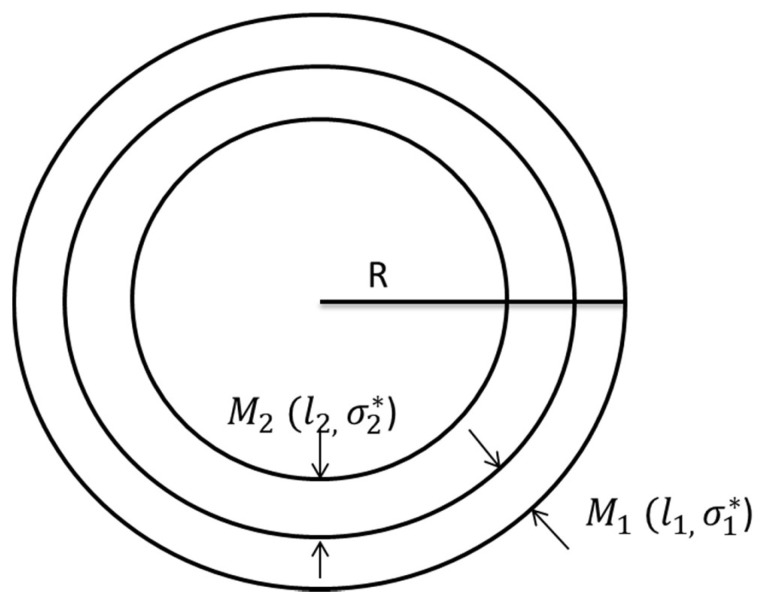
Equivalent cell membrane model.

The complicated calculation progress and the long computing time are caused by a large number of meshing elements around the thin cell membrane. The number of meshing elements could be reduced by enlarging the cell membrane thickness. However, changing the cell membrane thickness affects the conductivity spectrum of the cell suspensions. The aim of the proposed acceleration method is to keep the accuracy of the simulation result while enlarging the cell membrane thickness (reducing the number of elements). This acceleration method replaces the full-mesh model with an equivalent thicker cell membrane (reduce-mesh model) with an equivalent complex conductivity, as shown in Figure 3. The $M_{1}$ in Figure 3 is the full-mesh model cell membrane with normal thickness $l_{1}$. The $M_{2}$ in Figure 3 is the enlarged part of the reduce-mesh model cell membrane with thickness $l_{2}$, R is the radius of the cell model. The electrical parameter of the cell membrane $M_{2}$ is exactly the same with the intracellular fluid. The cell membrane $M_{1}$ and $M_{2}$ combine as one thicker equivalent cell membrane (reduce-mesh model cell membrane), and the thickness of the reduce-mesh model cell membrane is $l_{1}+l_{2}$. The number of meshing elements is reduced due to a thicker equivalent cell membrane. In this case, the factor v $={\left(1-\frac{d_{m}}{R}\right)}^{n}$ in Equation (1) can be rewritten as v $={\left(1-\frac{\left(l_{1}+l_{2}\right)}{R}\right)}^{n}$ to describe the Maxwell–Wagner effect in the equivalent model. According to the Maxwell–Wagner Equation (2), as the parameter P and $\varepsilon_{a}^{*}$ of the equivalent model are fixed, the dielectric behaviour of the suspension model $\varepsilon^{*}$ only depends on the dielectric property of the cell model $\varepsilon_{p}^{*}$. According to Equation (1), $\varepsilon_{p}^{*}$ is determined by $\varepsilon_{m}^{*}$ and the factor v. In order to retain the dielectric behaviour of the suspension model, the equivalent dielectric parameters of the equivalent cell membrane $\varepsilon_{m}^{*}$ can be calculated by Equations (14)–(18). With the equivalent complex conductivity, the behaviour of Maxwell–Wagner effect, which leads to β-dispersion remains the same.

The parameters of the cell membrane $M_{1}$ and $M_{2}$ in Figure 3 are:

(14) $$\sigma_{1}^{*}=\sigma_{1}+{j\omega \varepsilon }_{1}\varepsilon_{0}$$

(15) $$\sigma_{2}^{*}=\sigma_{2}+{j\omega \varepsilon }_{2}\varepsilon_{0}$$

where $\sigma_{1}^{*}$ and $\sigma_{2}^{*}$ are the complex conductivity of $M_{1}$ cell membrane and $M_{2}$ cell membrane in Figure 3, respectively. $\sigma_{1}$ and $\sigma_{2}$ are the conductivity of cell membrane $M_{1}$ and cell membrane $M_{2\;}$ in Figure 3, respectively. $\varepsilon_{1}$ and $\varepsilon_{2}$ are the relative permittivity of the cell membrane $M_{1}$ and cell membrane $M_{2,}$ respectively. Considering the two cell membranes $M_{1}$ and $M_{2}$ are connected as two electrolytes in series. The total impedance Z is:

(16) $$Z=Z_{1}+Z_{2}=\frac{l_{1}}{\sigma_{1}^{*}S_{1}}+\frac{l_{2}}{\sigma_{2}^{*}S_{2}}$$

where Z_1_ and Z_2_ are the impedance of the cell membrane $M_{1}$ and $M_{2}$, respectively. $\sigma_{1}^{*}$ and $\sigma_{2}^{*}$ are the complex conductivity of cell membrane $M_{1}$ and $M_{2}$ respectively. $l_{1}$ and $l_{2}$ are the thickness of the cell membrane $M_{1}$ and the cell membrane $M_{2}$ in Figure 3, respectively. $S_{1}$ and $S_{2}$ are the area of cell membrane $M_{1}$ and the cell membrane $M_{2}$, respectively. As $l_{1}$ and $l_{2}$ are negligible comparing with radius R, the area $S_{1}=S_{2}$. 

Then the equivalent complex conductivity can be derived as:

(17) $$Z=\frac{l_{1}}{\sigma_{1}^{*}S_{1}}+\frac{l_{2}}{\sigma_{2}^{*}S_{2}}=\frac{l_{1}\sigma_{2}^{*}+l_{2}\sigma_{1}^{*}}{\sigma_{1}^{*}\sigma_{2}^{*}}\;\frac{1}{S_{1}}=\frac{\left(l_{1}+l_{2}\right)}{\sigma_{e}^{*}}\;\frac{1}{S_{1}}$$

(18) $$\sigma_{e}^{*}=\frac{\left(l_{1}+l_{2}\right)\sigma_{1}^{*}\sigma_{2}^{*}}{l_{1}\sigma_{2}^{*}+l_{2}\sigma_{1}^{*}}$$

$\sigma_{e}^{*}$ is the complex conductivity of the equivalent cell membrane (reduce-mesh model cell membrane).

The replacement of the thin cell membrane with an equivalent thicker structure appears to have reflected an equivalent Maxwell–Wagner effect from the computation results. Theoretically, less contrasting EM properties between the boundary of two materials cause less Maxwell–Wagner effect (i.e., less charge building-up). At the same time, thicker materials between the two charged surfaces would mean the equivalent capacitance is smaller. These two effects would mean the electric field E = Q/C would be kept to a constant; this is possibly the reason why we can use the replacement to carry out the calculation of the dispersion caused by the MW effect. Equations (17) and (18) considered the complex conductivity, and therefore, the physics would be masked to a certain degree, but the principle can be explained as above.

### 2.5. Meshing Details

The suspension model shown in Figure 1 was discretised into three regions, cell membrane and intra and extracellular fluid, for meshing. The commercial software Comsol 5.0 was used for meshing. The convergent criterion was accelerated BICGS with an optimal precondition. The initial precondition was based on optimised initial guess: the final solution from the previous frequency [25,26,27,28,29,30]. As shown in Table 1, the minimum and maximum element sizes are set to be the same to maintain the meshing accuracy. The maximum element growth rate controls the maximum changing rate of the dimension of the element among the adjacent subdomains. The narrow factor controls the mesh density on the narrow region. Increasing the cell membrane thickness reduces the narrow regions. Therefore, the meshing density is larger in the cell model with smaller cell membrane thickness. Table 2 shows the number of meshing element in each specific region, including total suspension, cell membrane, intracellular fluid and extracellular fluid. In addition, a specific region surrounding the cell membrane was created to obtain the number of meshing elements around the cell membrane. It is obvious from Table 2 that the most meshing elements concentrate at the region around the cell membrane. The narrow region is reduced when the cell membrane thickness is enlarged, thus, the number of meshing elements is decreased when increasing cell membrane thickness.

## 3. Results and Discussion

### 3.1. D Single Spherical Cell Model

**Figure 4:**
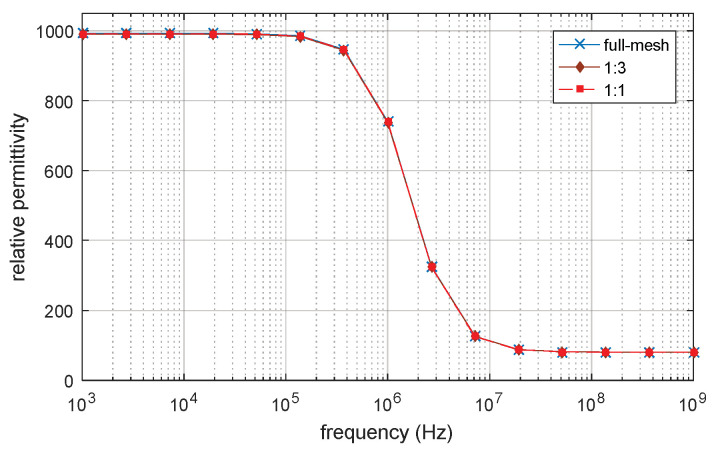
Permittivity of 2D single spherical cell model.

**Figure 5:**
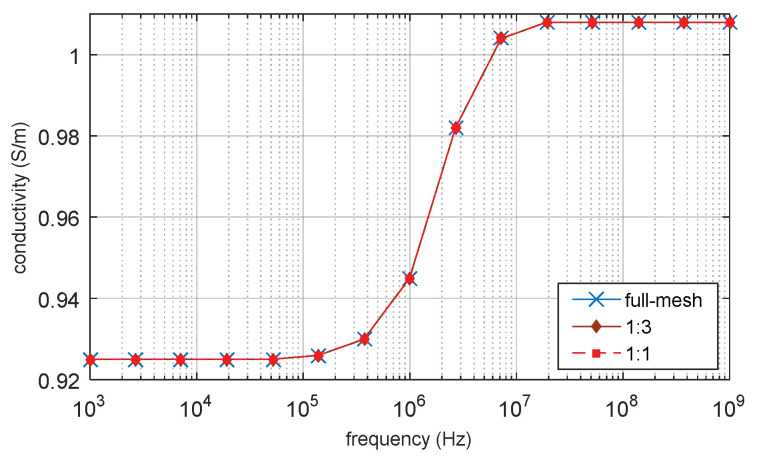
Of 2D single spherical cell model.

The full-mesh model cell membrane thickness is 5 nm. There are two simulations with equivalent thicker cell membrane (reduce-mesh model) thickness of 10 nm and 20 nm, respectively. The ratio between the full-mesh model cell membrane (cell membrane M_1 in Figure 3) thickness and the enlarged part of reduce-model cell membrane (cell membrane M_2 in Figure 3) thickness is 1:1 and 1:3, respectively. The simulation results are the progress of β-dispersion which reflects dispersions on relative permittivity and conductivity, as shown in Figure 4 and Figure 5. The main characteristics of β-dispersion are the dispersion frequency range and the magnitude of the relative permittivity and conductivity [27,30,31]. As shown in Figure 4, the dispersion frequency is ranging from 100 kHz to 10 MHz. The magnitude of relative permittivity at low frequency and high frequency are 960 and 80, respectively. The magnitude of conductivity at low frequency and high frequencies were 0.925 S/m and 1.04 S/m, respectively. This work was aimed at accelerating the computing progress with high accuracy, 15 sample points are selected in Figure 4 to describe the main feature of an entire β -dispersion and verify the accuracy of the acceleration method. The results of acceleration models are validated by the full-mesh model to verify the accuracy of the acceleration method. The errors between the acceleration model and full-mesh model at each point in Figure 4 and Figure 5 have been calculated. The calculated error and computing time for acceleration model and full-mesh model are shown in Table 3.

As shown Table 3, the number of elements of the full-mesh model can be reduced to 24% when the equivalent cell membrane (reduce-mesh model) is four times as the thickness of the full-mesh model cell membrane ($M_{1}$ cell membrane to $M_{2}$ cell membrane thickness ratio is 1:3). The computing time was reduced from 73 min to 13 min, with only a 0.2% error on the simulation result. The computing time of the reduce-mesh model is 18% of the full-mesh model. The spectroscopy of conductivity and relative permittivity is shown in Figure 4 and Figure 5, the results of the reduce-model and full-mesh model show the same magnitude over the same frequency range with an error less than 0.2%. The β dispersion of reduce-model and full-mesh model both starts at 100 kHz and both ends at 10 MHz. This shows that the reduce-mesh model can significantly accelerate the computing progress with only a tiny error on the result in the 2D-FEM spherical model simulation.

### 3.2. D Cell Deformation Model

This simulation is to verify that the acceleration method not only works on spherical cell model but also works on the deformation model (oval model).

**Figure 6:**
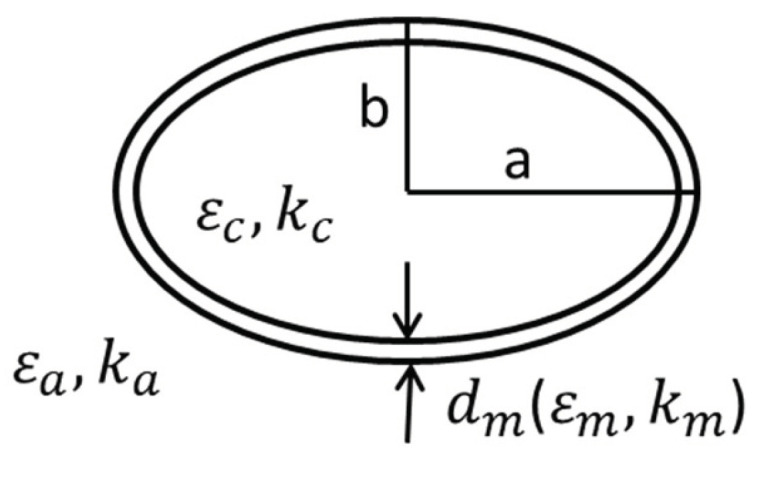
Deformation model.

**Figure 7:**
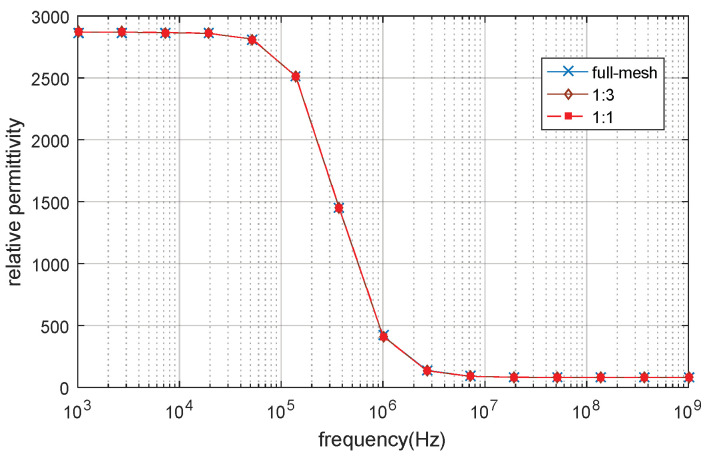
The relative permittivity of the 2D single deformation cell model.

**Figure 8:**
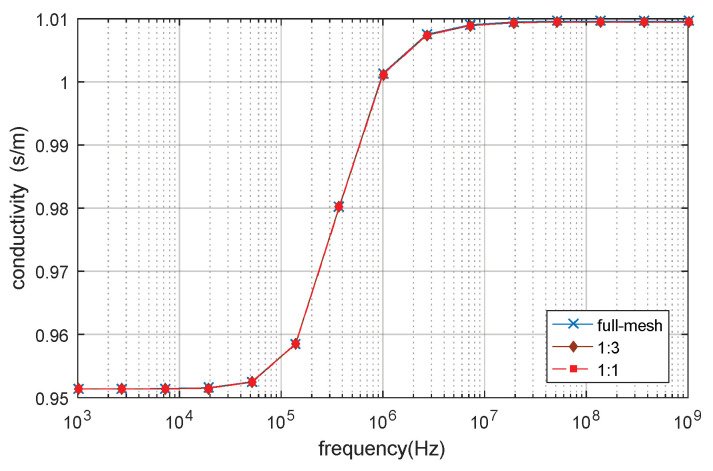
The conductivity of 2D single deformation cell model.

The deformation model is an oval model in order to simulation the cell deformation [28]. The electrical parameters of the oval model are the same as that of the custom spherical model. The model is shown in Figure 6, where a and b are the length of the semi-major and semi-minor axis, respectively. The length of the semi-major axis is set to be a = 12 μm and the length of the semi-minor axis is set to be b = 2 μm, $k_{a}$ and $k_{c}$ are the conductivity of the extracellular and intracellular fluid, respectively, $\varepsilon_{c}$ and $\varepsilon_{a}$ are the relative permittivity of the intracellular and extracellular fluid respectively, $\varepsilon_{m}$ is the relative permittivity of cell membrane, $d_{m}$ is the cell membrane thickness. The full-mesh model cell membrane thickness is still 5 nm and the electrical properties are calculated using equation 18. The simulation result of β dispersion is shown in Figure 7, Figure 8 and the parameters of the computing time and error are shown in Table 4.

The number of elements of full-mesh model can be reduced to 24.1% as shown in Table 4 and the computing time is reduced from 2h 24mins to 25mins. The simulation error between reduce-mesh model and full-mesh model is only 0.11%. As shown in Figure 7 and Figure 8, the results of the full-mesh model and reduce-mesh model exhibits β dispersion with tiny error in the same frequency range. The results show that the acceleration method is feasible to simulate not only the spherical model but also deformation model (oval model). The computing time is significantly reduced with acceptable error.

### 3.3. D Spherical Cell Model

**Figure 9:**
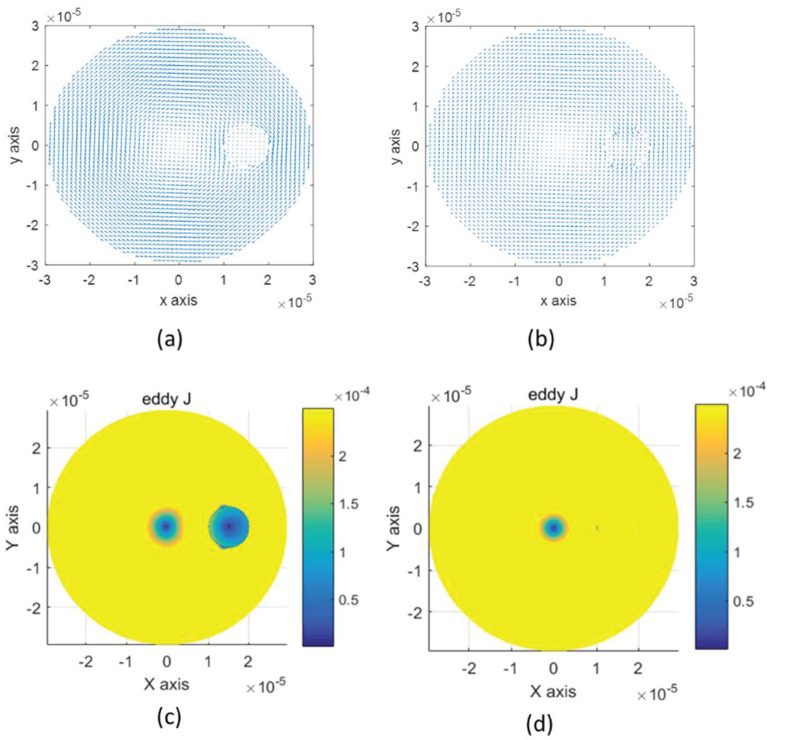
(**a**) Eddy current flow at low frequency 1 kHz. (**b**) Eddy current flow at high frequency 10 MHz. (**c**) Eddy current density at low frequency 1 kHz. (**d**) Eddy current density at high frequency 10 MHz.

The simulation model used for 3D FEM simulation is shown in Figure 2. 3-D single sphere cell model is used. The radius of the cell model is set to be 5 μm, and the cell membrane thickness is 5 nm. The top view of eddy current distributions at lower frequency 1 kHz and higher frequency 10 MHz are shown in Figure 9. The single-cell model blocks eddy currents at a lower frequency and becomes conductive at a higher frequency which meets the expectations of maxwell–wagner effect and other published works [9,11,15,29].

**Figure 10:**
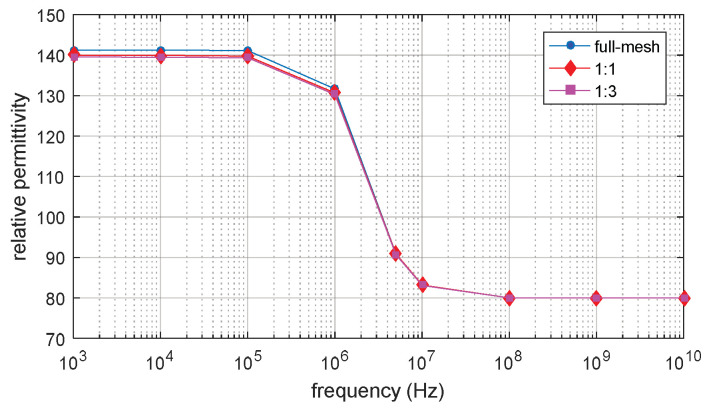
The relative permittivity of the 3D single spherical cell model.

**Figure 11:**
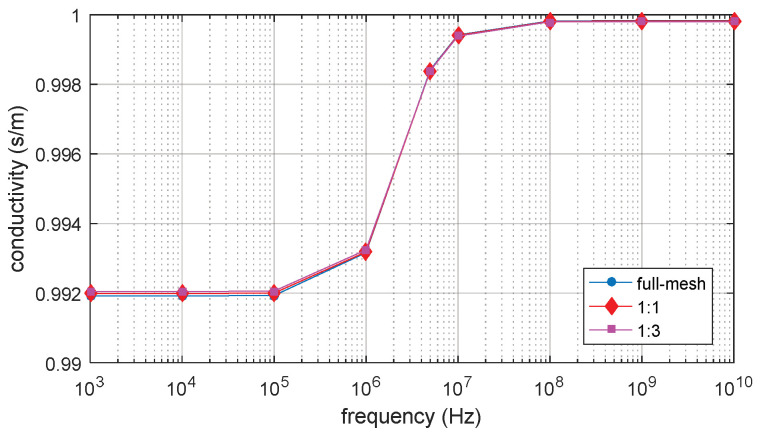
The conductivity of 3D single spherical cell model.

The full-mesh model cell membrane thickness is 5 nm. There are two reduce-models with equivalent thicker cell membrane (reduce-mesh model) thickness of 10 nm and 20 nm, respectively. The ratio between the full-mesh model cell membrane thickness and the reduce-mesh cell membrane thickness is 1:1 and 1:3, respectively. The relative permittivity and conductivity result are shown in Figure 10 and Figure 11. β dispersion can be observed from Figure 10 and Figure 11. The β dispersion of the full-mesh model and reduce-mesh model has an error of 2% on the magnitude. The frequency range of the β dispersion of full-mesh model and reduce-mesh model are the same, ranging from 100 kHz to 10 MHz. The results, including the number of elements, the computing time and error of full-mesh model and reduce-mesh model, are shown in Table 5. The number of elements of the full-mesh model can be reduced to 28%, as shown in Table 5, and the computing is reduced from 4 h 37 min to 71 min. The simulation error between reduce-mesh model and full-mesh model is 2%. The error is larger than the two-dimensional simulation result, but it is acceptable for β-dispersion simulations. The results show that the acceleration method is feasible to simulate a three-dimensional spherical model. The computing time is significantly reduced with acceptable error.

### 3.4. Experimental Validation by Contact-Electrode Measurement

The four-electrode measurement system is used to measure the impedance spectroscopy of biological samples. The electrode is contacted directly to the samples to measure the impedance over the samples using an impedance analyser. The measurement system work in the following way: the electrodes generate an input signal which applies an alternating electric field and current in the samples. By measuring the current and voltage across the samples, the conductivity and permittivity of the samples can be calculated. The impedance analyser used in this work is Solatron 1260, which is efficient and accurate over the frequency ranging from hundreds of hertz to 32 MHz. The effective area of the measurement electrodes is fixed to $S=2\;\mathrm{cm}\;*\;3\;\mathrm{cm}=6{\;\mathrm{cm}}^{2}$, and the distance between the electric field measuring electrodes is fixed to L=6.6 cm. The conductivity can then be calculated by σ=1/ρ=L/RS. R is the real part of the measured impedance, ρ is the resistivity. The measurement sample is fresh potato (obtained from a local market). This measurement is to validate the FEM simulation result. All FEM simulation parameters are set to be the realistic value obtained from the measurement.

**Figure 12:**
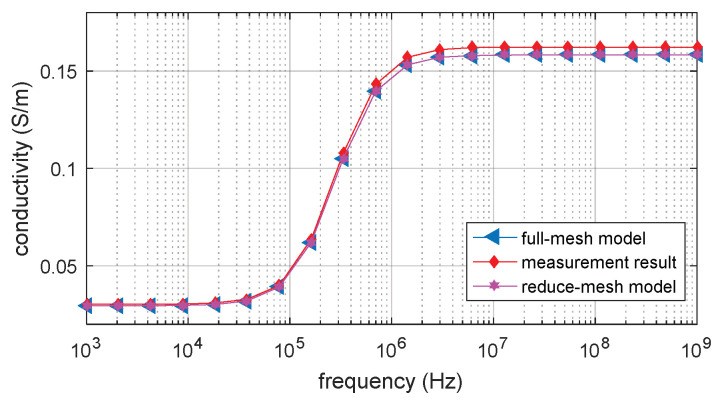
Impedance spectroscopy of FEM and measurement result.

In Figure 12, the FEM and measurement results show similar β -dispersion with the same dispersion frequency range. There is some error on the magnitude of the conductivity between the measurement result and the simulation result. However, the error is acceptable for the simulation approach of the measurement result. The conductivity of measurement and simulation result is low at a lower frequency, and the dispersion starts at around 50 kHz. The dispersion ends at around 2 MHz and the conductivity of measurement and simulation result is increased to 0.15 S/m. The spectroscopy curve is flat over the higher frequency range. The measurement result validated the full-mesh and reduce-mesh FEM simulation result and agreed with other published works [9,13,15,21,25,32].

### 3.5. Verification by Analytical Result

**Figure 13:**
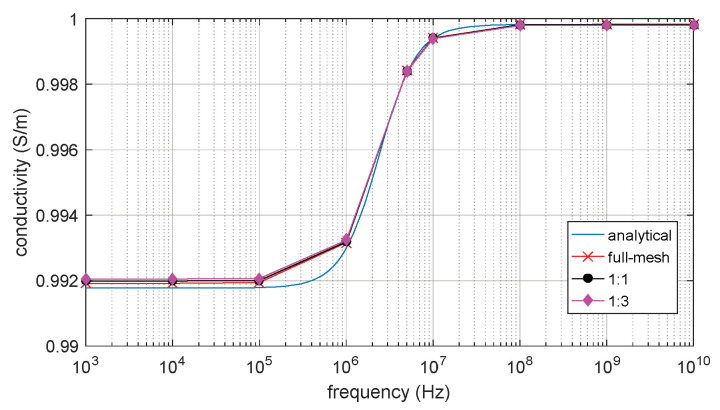
Calculated conductivity of the analytical solution and FEM simulation.

**Figure 14:**
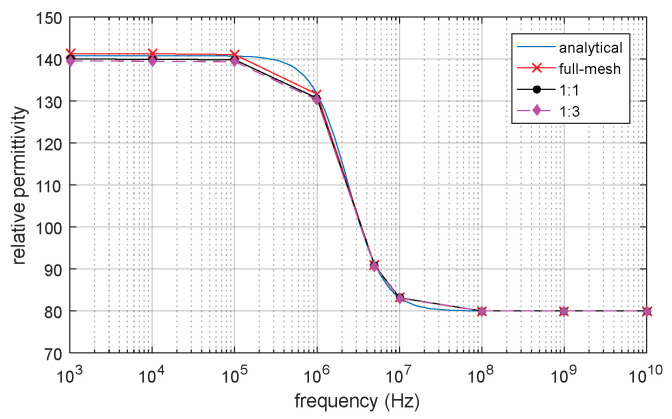
Calculated relative permittivity of the analytical solution and FEM simulation.

The analytical result can be calculated according to Maxwell–Wagner Equations (1) and (2). The analytical result is compared with the 3D finite element simulation result. So the factor v in equation (1) is defined as v $={\left(1-\frac{d_{m}}{R}\right)}^{3}$. According to Schwan [9], the impedance spectroscopy of the cell model can be described by Debye relaxation based on Equation (4). In Figure 13 and Figure 14, both analytical and FEM results show the same magnitude and frequency range of beta dispersion with an error less than 0.1%. The volume fraction of the cell is set to be the same as P = 3.5%. The magnitude and frequency range of β-dispersion on the full-mesh model and reduce-mesh model agree with the analytical result. The result validates that the three-dimension FEM simulation on cell suspension is accurate and the improved acceleration method is also validated. The proposed acceleration method can be applied to further FEM simulations on irregular shape cell models and thin shell models.

## 4. Conclusions

This paper proposed a method to accelerate FEM calculation with bio-cell models. The idea is to replace the thin cell membrane (full-mesh model) with an equivalent thicker cell membrane (reduce-mesh model). Then the number of meshing elements of the full-mesh model is reduced, and thus the computing time is reduced.

According to the simulation results, the reduce-mesh model can be used in both 2D and 3D FEM simulations. The amount of computing time is significantly reduced with an error no more than 0.5% in 2D simulation. The error on 3D simulation result is no more than 2%. All simulation results are validated by measurements and analytical results. 

The proposed acceleration method is validated to be fast and accurate on cell models and the acceleration method has potential for all other thin shell FEM models. 

## Figures and Tables

**Table 1:** Meshing information for acceleration model.

	Original Model Meshing Information	Acceleration Model Meshing Information $\left(l_{1}:l_{2}=1:1\right)$
Maximum element size (μm)	0.1	0.1
Minimum element size (μm)	0.01	0.01
Maximum element growth rate	1.20	1.20
Narrow factor	1	1

**Table 2:** The number of meshing element in different regions.

Regions	Original Model	Acceleration Model $\left(l_{1}:l_{2}=1:1\right)$
Extracellular fluid	32,814	15,668
Intracellular fluid	30,566	14,748
Cell membrane	9712	4856
Region around cell membrane	70,528	33,620
Total suspension	73,092	35,272

**Table 3:** Results of the 2D spherical cell model.

Thickness Ratio $\left(M_{1}/M_{2}\right)$	Number of Elements	Error	Computing Time (Minutes)
Original model	73,092	N/A	73
1:1	35,272	0.07%	28
1:3	17,342	0.2%	13

**Table 4:** Results of 2D Deformation Cell Model.

Thickness Ratio $\left(M_{1}/M_{2}\right)$	Number of Elements	Error	Computing Time
Original (a = 12, b = 2)	133,184	N/A	2 h 24 min
1:1	64,600	0.04%	62 min
1:3	32,097	0.11%	25 min

**Table 5:** Result of 3D Spherical Cell Model.

Thickness Ratio $\left(M_{1}/M_{2}\right)$	Number of Elements	Error	Computing Time
Original model	205,604	N/A	4 h 37 min
1:1	141,870	0.4%	3 h 5 min
1:3	57,934	2%	1 h 11 min

## References

[1] Gimsa J., Schnelle T., Zechel G., Glaser R. (1994). Dielectric spectroscopy of human erythrocytes: Investigations under the influence of nystatin. Biophys. J..

[2] Awayda M.S., Driessche W., Helman S.I. (1999). Frequency-dependent capacitance of the apical membrane of frog skin: Dielectric relaxation processes. Biophys. J..

[3] Mukhopadhyay S.C., Gooneratne C.P. (2007). A novel planar-type biosensor for noninvasive meat inspection. IEEE Sens. J..

[4] Jaffrin M.Y., Morel H. (2008). Body fluid volumes measurements by impedance: A review of bioimpedance spectroscopy (BIS) and bioimpedance analysis (BIA) methods. Med. Eng. Phys..

[5] Morimoto T., Kimura S., Konishi Y., Komaki K., Uyama T., Monden Y., Kinouchi D.Y., Iritani D.T. (1993). A study of the electrical bio-impedance of tumors. J. Investig. Surg..

[6] Kerner T.E., Paulsen K.D., Hartov A., Soho S.K., Poplack S.P. (2002). Electrical impedance spectroscopy of the breast: Clinical imaging results in 26 subjects. IEEE Trans. Med. Imaging.

[7] Romsauerova A., McEwan A., Horesh L., Yerworth R., Bayford R.H., Holder D.S. (2006). Multi-frequency electrical impedance tomography (EIT) of the adult human head: Initial findings in brain tumours, arteriovenous malformations and chronic stroke, development of an analysis method and calibration. Physiol. Meas..

[8] Schwan A., Herman P. (1957). Electrical properties of tissue and cell suspensions. Adv. Biol. Med. Phys..

[9] Schwan A., Herman P. Electrical properties of tissues and cell suspensions: Mechanisms and models. Proceedings of the 16th Annual International Conference of the IEEE Engineering in Medicine and Biology Society.

[10] Damez J.L., Clerjon S., Abouelkaram S., Lepetit J. (2007). Dielectric behavior of beef meat in the 1–1500 kHz range: Simulation with the Fricke/Cole–Cole model. Meat Sci..

[11] Stubbe M., Gimsa J. (2015). Maxwell’s Mixing Equation Revisited: Characteristic Impedance Equations for Ellipsoidal Cells. Biophys. J..

[12] Asami K., Tetsuya H., Naokazu K. (1980). Dielectric approach to suspensions of ellipsoidal particles covered with a shell in particular reference to biological cells. Jpn. J. Appl. Phys..

[13] Pauly H., Schwan H.P. (1959). Impedance of a suspension of ball-shaped particles with a shell; a model for the dielectric behavior of cell suspensions and protein solutions. Z. Naturforsch..

[14] Tuncer E., Stanisław M., Gubański C., Nettelblad B. (2001). Dielectric relaxation in dielectric mixtures: Application of the finite element method and its comparison with dielectric mixture formulas. J. Appl. Phys..

[15] Asami K. (2006). Dielectric dispersion in biological cells of complex geometry simulated by the three-dimensional finite difference method. J. Phys. D Appl. Phys..

[16] Mejdoubi A., Brosseau C. (2006). Finite-element simulation of the depolarization factor of arbitrarily shaped inclusions. Phys. Rev. E.

[17] Takashima S., Asami K., Takahashi Y. (1988). Frequency domain studies of impedance characteristics of biological cells using micropipet technique. Biophys. J..

[18] Fricke H. (1953). The Maxwell-Wagner dispersion in a suspension of ellipsoids. J. Phys. Chem..

[19] Jin J.M. (2015). The Finite Element Method in Electromagnetics.

[20] Bíró O. (1999). Edge element formulations of eddy current problems. Comput. Methods Appl. Mech. Eng..

[21] O’Toole M.D., Marsh L.A., Davidson J.L., Tan Y.M., Armitage D.W., Peyton A.J. (2015). Non-contact multi-frequency magnetic induction spectroscopy system for industrial-scale bio-impedance measurement. Meas. Sci. Tech..

[22] Georgii J., Rudiger W. (2010). A streaming approach for sparse matrix products and its application in Galerkin multigrid methods. Electron. Trans. Numer. Anal..

[23] Fritschy J., Horesh L., Holder D.S., Bayford R.H. (2005). Using the GRID to improve the computation speed of electrical impedance tomography (EIT) reconstruction algorithms. Physiol. Meas..

[24] Lu M.Y., Anthony P., Yin W.L. (2017). Acceleration of Frequency Sweeping in Eddy-Current Computation. IEEE Trans. Magn..

[25] Gheorghiu E. (1996). Measuring living cells using dielectric spectroscopy. Bioelectrochem. Bioenerg..

[26] Sekine K. (2000). Application of boundary element method to calculation of the complex permittivity of suspensions of cells in shape of D∞ h symmetry. Bioelectrochemistry.

[27] Beving H., Eriksson L.E.G., Davey C.L., Kell D.B. (1994). Dielectric properties of human blood and erythrocytes at radio frequencies (0.2–10 MHz); dependence on cell volume fraction and medium composition. Eur. Biophys. J..

[28] Di Biasio A., Cametti C. (2007). Effect of shape on the dielectric properties of biological cell suspensions. Bioelectrochemistry.

[29] Saville D.A., Bellini T., Degiorgio V., Mantegazza F. (2000). An extended Maxwell–Wagner theory for the electric birefringence of charged colloids. J. Chem. Phys..

[30] Davis L.C. (1992). Polarization forces and conductivity effects in electrorheological fluids. J. Appl. Phys..

[31] Zhou Y.Y., Wang A.P., Zhou P., Wang H., Chai T.Y. (2020). Dynamic performance enhancement for nonlinear stochastic systems using RBF driven nonlinear compensation with extended Kalman filter. Automatica.

[32] Tang J.W., Yin W.L., Lu M.Y. (2020). Bio-impedance spectroscopy for frozen-thaw of bio-samples: Non-contact inductive measurement and finite element (FE) based cell modelling. J. Food Eng..

